# Excess mortality associated with the COVID-19 pandemic during the 2020 and 2021 waves in Antananarivo, Madagascar

**DOI:** 10.1136/bmjgh-2023-011801

**Published:** 2023-07-26

**Authors:** Joelinotahiana Hasina Rabarison, Jean Marius Rakotondramanga, Rila Ratovoson, Bruno Masquelier, Anjaraso Maharavo Rasoanomenjanahary, Anou Dreyfus, Andres Garchitorena, Fidisoa Rasambainarivo, Norosoa Harline Razanajatovo, Soa Fy Andriamandimby, C Jessica Metcalf, Vincent Lacoste, Jean-Michel Heraud, Philippe Dussart

**Affiliations:** 1 Institut Pasteur de Madagascar, Antananarivo, Madagascar; 2 UMMISCO, Bondy, France; 3 Universite Catholique de Louvain Centre de recherche en demographie et societes, Louvain la neuve, Belgium; 4 Bureau Municipal d’Hygiène, Commune Urbaine d’Antananarivo, Antananarivo, Madagascar; 5 UMR 224 MIVEGEC, IRD, Montpellier, France; 6 Department of Ecology and Evolutionary Biology, Princeton University, Princeton, New Jersey, USA; 7 Mahaliana Labs SARL, Antananarivo, Madagascar; 8 Institut Pasteur de Dakar, Dakar, Senegal

**Keywords:** COVID-19, epidemiology, public health, respiratory infections

## Abstract

**Introduction:**

COVID-19-associated mortality remains difficult to estimate in sub-Saharan Africa because of the lack of comprehensive systems of death registration. Based on death registers referring to the capital city of Madagascar, we sought to estimate the excess mortality during the COVID-19 pandemic and calculate the loss of life expectancy.

**Methods:**

Death records between 2016 and 2021 were used to estimate weekly excess mortality during the pandemic period. To infer its synchrony with circulation of SARS-CoV-2, a cross-wavelet analysis was performed. Life expectancy loss due to the COVID-19 pandemic was calculated by projecting mortality rates using the Lee and Carter model and extrapolating the prepandemic trends (1990–2019). Differences in life expectancy at birth were disaggregated by cause of death.

**Results:**

Peaks of excess mortality in 2020–21 were associated with waves of COVID-19. Estimates of all-cause excess mortality were 38.5 and 64.9 per 100 000 inhabitants in 2020 and 2021, respectively, with excess mortality reaching ≥50% over 6 weeks. In 2021, we quantified a drop of 0.8 and 1.0 years in the life expectancy for men and women, respectively attributable to increased risks of death beyond the age of 60 years.

**Conclusion:**

We observed high excess mortality during the pandemic period, in particular around the peaks of SARS-CoV-2 circulation in Antananarivo. Our study highlights the need to implement death registration systems in low-income countries to document true toll of a pandemic.

**WHAT IS ALREADY KNOWN ON THIS TOPIC**

- Sub-Saharan countries lag behind the rest of the world in vital statistics leading to the difficulty of collecting information on annual deaths.

**WHAT THIS STUDY ADDS**

- Our study is one of the firsts conducted in sub-Sahara Africa that made use of a local death notification system to assess the impact of COVID-19 pandemic on mortality and life expectancy in the general population.
- The mortality burden during the COVID-19 pandemic in Antananarivo has been much higher than the official count of COVID-19-related deaths for the entire country.

**HOW THIS STUDY MIGHT AFFECT RESEARCH, PRACTICE OR POLICY**

- Our study highlights the importance of local sources of death notification to better estimate mortality in the context of an outbreak or a pandemic.

## Introduction

Mortality has declined substantially in Madagascar over the past few decades, with life expectancy increasing by almost 15 years (from 52 to 66 years) between 1990 and 2022.^1^ This progress was largely driven by a rapid decline in under-five mortality, which was reduced by two-thirds over the same period.^2^ This gain is however fragile, in a country like Madagascar characterised by persistent high rates of poverty, political instability, a weak health system, rapid population growth and scarcity of resources.^3 4^ The recent epidemics of pneumonic plague in 2017 and measles in 2018–19 provide vivid examples of the precariousness of the health situation in the country.^5 6^ The COVID-19 pandemic might have jeopardised the life expectancy gains achieved over the last decades. In many low-income and middle-income countries (LMICs), in particular in sub-Saharan Africa, deaths associated with the COVID-19 remain difficult to estimate, however, mainly due to the absence of a comprehensive and efficient civil registration and vital statistics system. According to estimates by WHO, when compared with the mortality expected in the absence of the pandemic, COVID-19 caused directly and indirectly, 25 582 additional deaths during the 2020–21 period in Madagascar.^7^ This would represent an increase of approximately 14% over the estimated annual number of deaths in the country.^1^ However, as of 31 December 2021, the Malagasy Ministry of Health (MoH) notified 1067 deaths due to COVID-19 for the entire country.^8^ This discrepancy between WHO estimates and the Malagasy MoH deaths could be explained by the fact that the MoH only notified COVID-19 laboratory-confirmed deaths, and left indirect deaths unaccounted for. Moreover, a considerable proportion of deaths occurred outside the healthcare system and were never reported in the official statistics. Consequently, the total number of direct and indirect deaths due to the COVID-19 pandemic is unknown in Madagascar. Epidemiological models such as the WHO model are based on a number of covariates including the reported COVID-19 mortality rate and the COVID-19 positivity rate. But these official statistics can themselves be heavily biased due to limited testing capacity (max 1000 tests/day), insufficient staff to conduct contact tracing and changes in the testing policy during the course of the pandemic.^9^ There are also large uncertainties around prepandemic baseline mortality rates due to the incompleteness of death registration systems in LMICs. For example, in Guinea-Bissau, a seroprevalence study in the capital showed that the official number of PCR-confirmed COVID-19 cases greatly underestimated the prevalence of COVID-19 during the pandemic.^10^ In Zambia, most deaths due to COVID-19 were detected in the community outside of hospitals and were not tested before death.^11^

Compared with other African countries, the situation of Madagascar is atypical since the capital city of Antananarivo has a relatively unique system of death notification in place, covering both hospital and community deaths, located at the Bureau Municipal d’Hygiène (BMH) of Antananarivo. An evaluation of this system before the pandemic concluded that high completeness of registration had been achieved over the last decades, with accurate data on the underlying diseases as established by physicians visiting relatives of the deceased or certifying deaths in healthcare facilities.^12^ The available registers cover the period 1976–2021 and have previously been analysed to document a mortality crisis due to the resurgence of malaria and an urban famine in the mid-80s,^13^ the excess mortality during the H1N1 pandemic in 2009^14^ and the measles outbreak in 2018–19.^15^

The objectives of our study were to (i) leverage this death notification system to reconstruct recent mortality trends covering the years 2020 and 2021 in Antananarivo and (ii) assess the effects of the COVID-19 pandemic on mortality in the capital city of Madagascar.

## Methods

### Study site and data sources

Since 2016, the National Influenza Centre located at the Institut Pasteur de Madagascar (IPM) has improved the death surveillance system at BMH. This office covers five of the six central districts of Antananarivo city, corresponding to the district of Antananarivo-Renivohitra with a total population of 1.14 million inhabitants according to the 2018 census (online supplemental method S1).^16^ The BMH is responsible for delivering and storing death certificates. To date, nine physicians are responsible for home-visits to establish a death certificate indicating the cause of death. The assignment of a cause of death is based on information provided by the family and available medical documentation. If the death occurred at the hospital, the family presents a death certificate signed by a clinician to the BMH. This hospital death certificate also indicates the presumed cause of death. For each death, the register includes the name of the deceased, date of birth, date of death, gender, address, place of death (hospital or home) and cause of death coded according to the International Classification of Diseases (ICD) (currently using the ICD 10th revision). In 2020 and 2021, COVID-19 deaths were coded as ‘B97.2’ or ‘U07’, following regulations set by WHO. For deaths that occurred in 2020 and 2021 within hospitals, the certificate also indicates the result of the COVID-19 test. Deaths at home were not tested but diagnosed according to clinical assessment by physicians and according to the epidemiological context. For the study period, we considered all death certificates stored at the BMH between 2016 and 2021. We also resorted to tabulations of deaths by age and sex from 1990 to 2015 to reconstruct trends in mortality over the period prepandemic.^12^ Using the address of the deceased in the city of Antananarivo from registers of the BMH, we could restrict the analysis to residents of five of the six districts. The sixth district, Ambohimanarina, does not fall in the area covered by the BMH for historical reasons (online supplemental method S1).

10.1136/bmjgh-2023-011801.supp1 Supplementary data

### Calculation of weekly excess deaths

To calculate weekly excess deaths, we considered calendar weeks (1–52nd) and assessed excess mortality in 2020 and 2021 as the percentage difference between the reported and the average expected number of deaths of the same week, using the relative change measure based on Farrington surveillance algorithm.^17^ First, the expected deaths were derived from the period 2016 to 2019, and the corresponding relative change was computed as the difference between the reported count and the upper bound of the 95% CI:

$$Relativechange\left(\%\right)=\;\frac{\left\{Reporteddeaths\right\}-\left\{Upperboundofexpecteddeaths\right\}}{\left\{Upperboundofexpecteddeaths\right\}}*100$$

A relative change of 100%, in a given week in 2020 (or 2021), would mean that the death count for that week was double from the expected death count. Based on the upper bound of the baseline average weekly deaths during 2016–19, excess deaths were then assessed by considering weeks where relative change values were associated with significant positive excess of deaths. In order to classify these relative changes of excess deaths, their estimated values were split in <50% and ≥50% changes.

Second, two sensitivity analyses were performed: (1) to assess the appropriateness of using the relative change in deaths based on Farrington surveillance algorithm instead of the simpler method of relative change (P-scores), which may alter the estimate of excess mortality (online supplemental method S2)^18^ and (2) to remove the effects of the 2018–19 measles outbreak, which could have induced large excess mortality in the capital, especially among children and youth.^6^ To do so, for the second sensitivity analysis, weekly deaths were estimated to be the same as historical averages for the same week: using 2016–17 historical data for the October–December 2018 outbreak, and 2016–18 historical data for the January–March 2019 outbreak. Then, the relative changes—as described above—were recalculated considering these adjustments for the baseline years, 2016–19.

### Cross-wavelet analyses

Cross-wavelet analyses were used to successively investigate associations between the time series of all-cause deaths over COVID-19 deaths (nationwide),^8^ and of SARS-CoV-2 positivity rate at the IPM laboratory^9^ over all-cause deaths (in Antananarivo). This method is useful for finding the frequency correlation between two non-stationary time series, combining time-frequency analyses and the statistical significance level of the relationships between lead and lag phases.^19^ It allows to characterise whether (i) changes in one time series follows or precedes similar changes in the other by a certain amount of time (ie, lead or lag), (ii) similar changes in both time series occur at the same time (ie, synchronous) or (iii) there is no association between the timing of changes in each of the time series. Here, the cross-wavelet analysis was performed twice for scaled time series according to each pair of comparisons of 2020–21 data: all-cause deaths in Antananarivo versus the registered COVID-19-related deaths in the whole country,^8^ and the SARS-CoV-2 positivity rate obtained from the virology unit of the IPM^9^ versus the all-cause deaths in Antananarivo. Using an appropriate combination of parameters, as detailed in the online supplemental method S3 (windows for time and period (scale) smoothing),^19^ these analyses can be used to infer the synchrony and association between two time series across time and period, providing wavelet coherence (the equivalent of the coefficient of determination in a statistical model) and phase-difference (angle).^20^ Further details about the rescaled data and the wavelet analyses coherence and phase-difference can be found in the online supplemental method S3.

### Calculation of trends in life expectancy

To construct annual life tables, the populations exposed to the risk of dying were reconstructed from the last three censuses of 1976, 1993 and 2018, assuming a constant rate of growth for each age segment, with growth rates estimated from the two intercensal periods and applied respectively to the periods 1975–1993 and 1993–2021. To calculate the life expectancy loss in 2020 and 2021 due to the COVID-19 pandemic, we projected mortality rates using the model by Lee and Carter and extrapolated the trends observed prepandemic, over the period 1990–2019.^21^ This model uses a singular value decomposition method to synthesise a matrix of logged age-specific mortality rates into three components: (1) an index $k_{t}$ that reflects the level of mortality (all ages) and varies over time, (2) a general age schedule of mortality ($a_{x}$) and (3) a set of constants ($b_{x}$) that capture which rates change most rapidly in response to a change in $k_{t}$:

This model is widely used for mortality projections. The $k_{t}$ index is extrapolated using an autoregressive integrated moving average process, while the other two components ($a_{x}$ and $b_{x}$) are held constant; and $e_{xt}\;$ is the residual at age x and time t. The difference between the observed life expectancy in 2020 and 2021 and the predicted values for these 2 years provided the number of years of life expectancy lost due to the pandemic, and prediction intervals around the life expectancy allowed us to establish whether the trend was significantly deviating from the recent past.

Second, differences in life expectancy at birth between 2019 and 2020–21 are an algebraic function of the differences in the underlying age-specific and cause-specific mortality rates.^22^ To compute the contribution of each age group and categories of causes of death to the life expectancy difference, we used a decomposition method designed by Arriaga.^23^ We first decomposed the difference by age. Then, assuming that the distribution of deaths by cause is constant within each age group, we further disaggregated the contribution within each age group by cause of death, isolating the COVID-19 deaths from all other causes.

All calculations were performed using R statistical software.^24^ WaveletComp R package was used to perform cross-wavelet coherence analyses.^20^

### Patient and public involvement

Patients and/or the public were not involved in the design, or conduct, or reporting, or dissemination plans of this research.

## Results

### Temporal trends of crude all-cause mortality in Antananarivo

**Figure 1:**
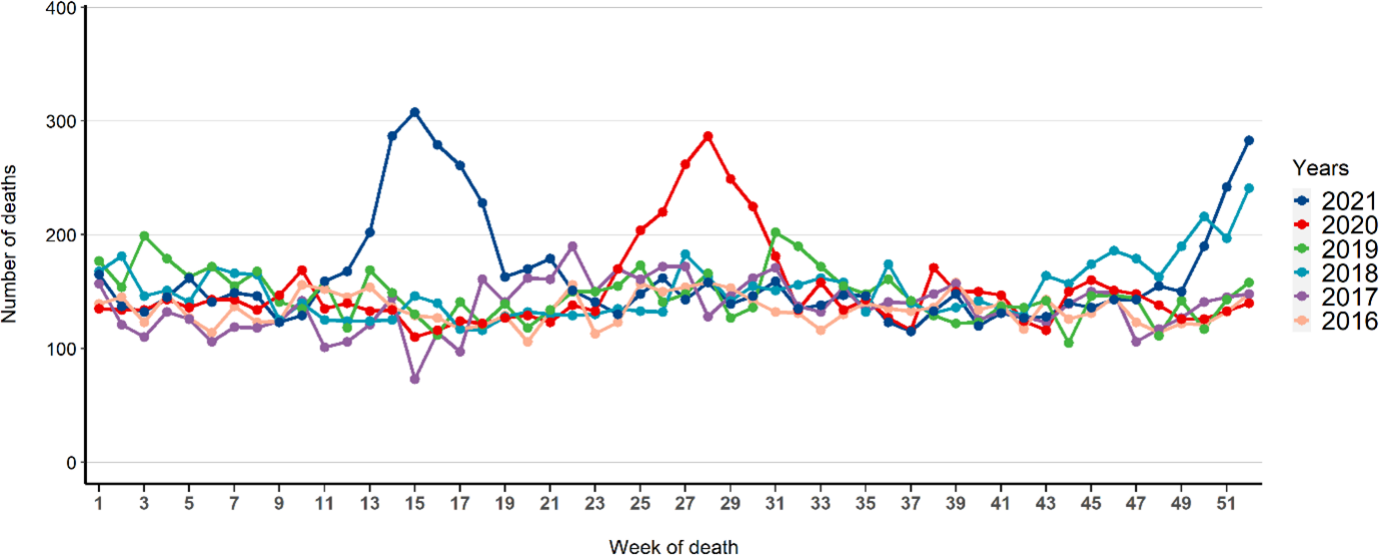
Temporal trends of all-cause crude mortality from 2016 to 2021 in the five central districts of Antananarivo (n=45 959).

A total of 45 959 death records, notified to the BMH, were included in our study from 2016 to 2021. Death records averaged 143 (range=118–174) deaths per week during the four prepandemic years (2016–19) considered as our baseline period. Before the COVID-19 pandemic period, waves with higher numbers of crude deaths were recorded at the end of 2018, reaching >200 weekly deaths during three consecutive weeks. The excess deaths observed in 2018 could be associated with the measles outbreak that affected the city during the last quarter of 2018.^6^ During the three successive waves of COVID-19, we observed higher peaks of deaths than in previous years, with >200 deaths in weeks 25–30 of 2020 and weeks 13–18 of 2021 as well as in the last weeks of 2021 (51 and 52) (figure 1 and online supplemental tables S1 and S2).

### Excess mortality during the 2020–21 waves of COVID-19 infection in Antananarivo

**Figure 2:**
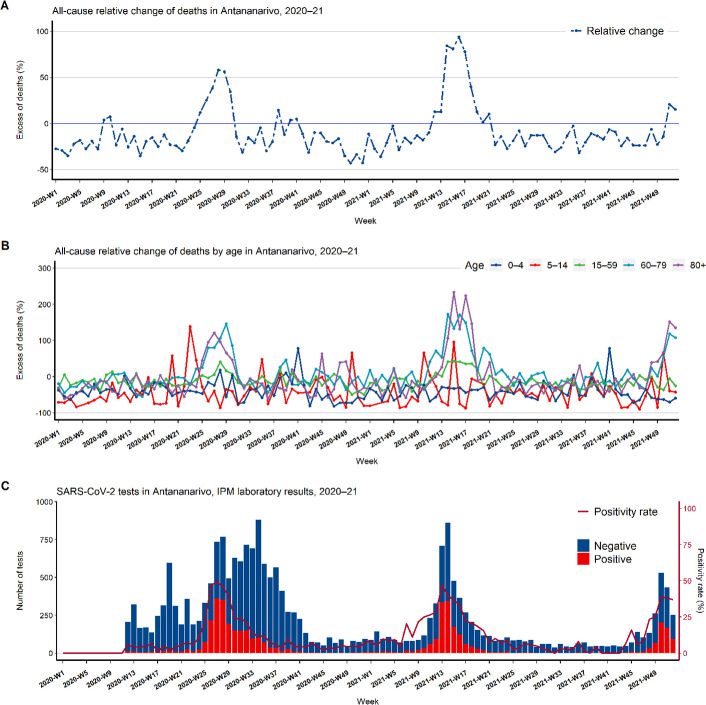
Weekly temporal trends, in five districts of Antananarivo from 2020 to 2021. (A) All-cause excess mortality using 2016–19 crude deaths as baseline years. (B) All-cause excess mortality by age group using 2016–19 crude deaths as baseline years (no death record was observed for age group 5–14 years during 1 week in 2020 and 2 weeks in 2021). (C) SARS-CoV-2 test results and positivity rates (data from the Institut Pasteur de Madagascar (IPM) for Antananarivo).^9^ Supplementary materials representing weekly all-cause deaths by age group (online supplemental table S1) and all-cause excess mortality by sex (online supplemental figure S2) support this figure.

A statistically significant increase in all-cause excess mortality was observed during 23 weeks in 2020 and 2021. During these weeks, we recorded 217 deaths per week on average, representing a 55.0% increase compared with 140 deaths per week over the same weeks during the baseline period (figure 2 and table 1). A total of 1179 all-cause excess deaths were assessed considering only significant relative change measures during the waves of COVID-19 in 2020 and 2021 in Antananarivo (table 1 and figure 2). Excess mortality of <50% was recorded during 9 and 8 weeks, corresponding to 244 and 222 excess deaths, respectively, in 2020 and 2021. In addition, excess deaths of 50% or greater, totalizing 195 and 518 deaths, were detected during 2 and 4 weeks in 2020 and 2021, respectively (table 1 and figure 2A). Normalised by the population size of the five districts of Antananarivo based on the general population census,^16^ the number of excess deaths were 38.51 and 64.91 per 100 000 inhabitants in 2020 and 2021, respectively. It is noteworthy that the positivity rate of SARS-CoV-2 virus was >25% during the same periods of the observed excess deaths with a peak reached 1 week before the peak of excess deaths in 2020 and 2021. Excess mortality was observed predominantly in adults aged 15–59 years and older adults aged 60 years and above. For these groups, the excess deaths seemed to increase with age during the 2020 and 2021 waves of SARS-CoV-2 positivity (figure 2B and online supplemental figure S2). In children and adolescents <15 years, there were very few weeks with excess deaths.

**Table 1:** Estimated all-cause excess mortality in 2020–21 using relative change and based on 2016–19 baseline of crude deaths data

Year	Relative change level (%)	Nb. of weeks of excess	Expected deaths (2016–19)		Reported deaths (2020–21)		Sum excesses death
			Mean	Range (minimum-maximum)	Mean	Sum	
2020	<50	9	144	128–164	188	1695	244
2020	≥100	2	148	142–154	268	536	195
2021	<50	8	141	123–174	204	1635	222
2021	≥100	4	125	118–139	284	1135	518
Total		23	140	118–174	217	5001	1179

Nb, number.

Of note, by adjusting deaths during measles outbreaks (October 2018–March 2019)^6^ from our baseline years, a higher all-cause excess mortality was estimated during waves of COVID-19 in both 2020 and 2021 in Antananarivo: totalling 1500 deaths during 32 weeks (online supplemental figure S3 and online supplemental table S3). Furthermore, by avoiding thresholds in our calculation, all-cause excess mortality was observed during 51 weeks in 2020 (from 1% to 87%) and 2021 (from 2% to 158%)—totalling 2111 deaths and estimated at over 200% for the elderly (80+ years) for each wave (online supplemental figure S4).

### Cross-wavelet coherence of SARS-CoV-2 positivity rate and death rate

**Figure 3:**
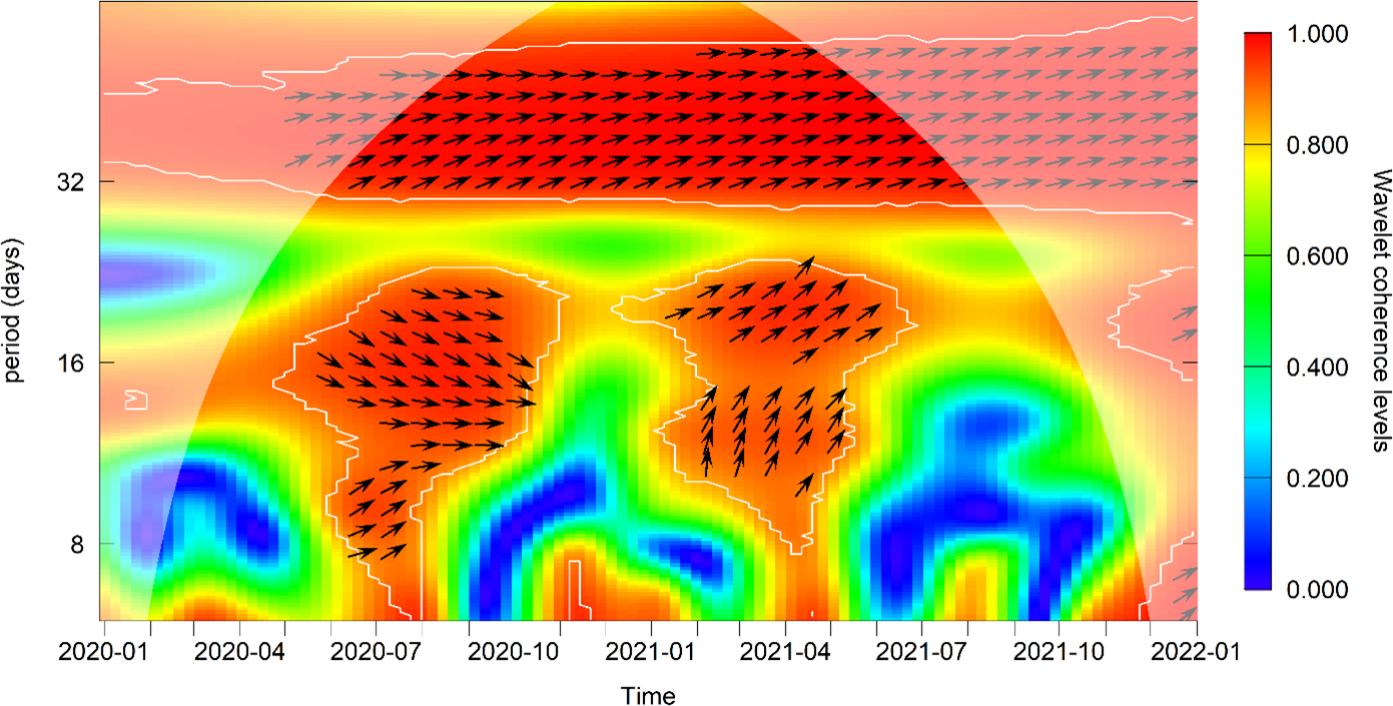
Cross-wavelet coherence and phase-difference of the SARS-CoV-2 positivity rate and all-cause death rate in Antananarivo. These two time series were in phase from June 2020 to September 2021 (phase-differences between 0 and $\frac{\pi }{2}$). During the first wave of COVID-19 (June 2020), the death rate seemed to lead the SARS-CoV-2 positivity rate (phase-differences $-\frac{\pi }{2}$ and 0). In the second wave (February 2021–May 2021), SARS-CoV-2 positivity rate significantly led the death rate (phase-differences between 0 and $\frac{\pi }{2}$). The significant coherence level is ranked from blue (0.0) to red (1.0) colour scale, and statistical significance is marked by the white contour plots, when p<0.05. Supplementary materials representing all-causes deaths (online supplemental figure S1B) and SARS-CoV-2 tests positivity (online supplemental figure S1D), also online supplemental method S3 support the interpretation of this figure.

SARS-CoV-2 test positivity rate by the IPM laboratory and all-cause death rate in Antananarivo, were in phase from June 2020 to September 2021 (online supplemental figure S1A, S1B, S1C, S1D); this was associated with a significant coherence level of ≥0.8 (p<0.05), showing that changes in SARS-CoV-2 test positivity rate over time occurred at the same time as equivalent changes in all-cause deaths in Antananarivo during the first wave of COVID-19 (figure 2C). The death rate seemed to slightly precede the SARS-CoV-2 test positivity rate during the growth phase of the first wave of COVID-19, in June 2020.^9^ However, in the second wave of COVID-19, from February 2021 to May 2021, there was a significant lead in the SARS-CoV-2 positivity rate on the death rate (figure 3). In addition, in the first half of 2020, all-cause deaths in Antananarivo and national COVID-19 deaths were not in synchrony (ie, these two time series were out of phase); and the weekly national COVID-19 deaths preceded the Antananarivo deaths (online supplemental figure S3). However, from the second semester of 2020 to September 2021, the all-cause deaths in Antananarivo were mainly in phase and preceded the national COVID-19 deaths, with a significant coherence level of ≥0.8 (p<0.05), showing that changes in the weekly national COVID-19 deaths occurred at the same time as equivalent changes in all-cause deaths in Antananarivo from second semester of 2020 to September 2021. Additional result interpretations can be found in online supplemental method S3 and online supplemental figure S5.

### Changes and differences in life expectancy

**Figure 4:**
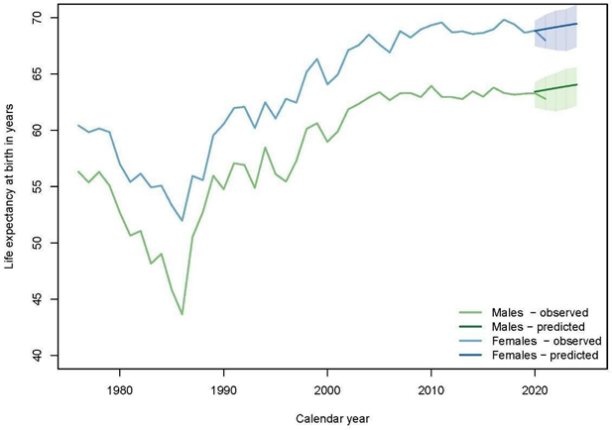
Observed and predicted life expectancy at birth in Antananarivo (1976–2021) for males (green) and females (blue). We projected to 2020–24, the observed life expectancy at birth and the predicted (bold) for both sexes based on the rates measured from 1990 to 2019.

For both sexes, life expectancy at birth declined in 2021, compared with the recent past, but the year 2020 was in line with earlier years (figure 4). Male life expectancy has shown very little progress in the capital since 2004, hovering around 63–64 years. Projections (without COVID-19) placed the estimates for 2020 and 2021 at 63.4 and 63.6 years, respectively, compared with the observed values of 63.3 and 62.8. The life expectancy has thus been reduced by 0.1 and 0.8 years for men in 2020 and 2021, respectively. For women, progress has also been very modest since the mid-2000s, with life expectancy appearing to plateau at around 68–69 years. Projections were 68.8 years in 2020 and 69.0 years in 2021, compared with 68.9 and 68.0 observed in the context of the pandemic. The levels are therefore virtually the same in 2020, while there is a 1-year drop in life expectancy in 2021. However, for both sexes, the observed drop in 2021 remains within the CIs around the projections, so life expectancy reduction associated with the COVID-19 is not statistically significant. The impact represented by COVID-19 on life expectancy was much smaller than the decline in life expectancy observed in the mid-1980s due to a resurgence of malaria and malnutrition.^13^

Table 2 displays the contributions by age to the differences in life expectancy between the observed life tables and the counterfactual life tables (based on extrapolating prepandemic rates). The contributions by age sum up to the difference in life expectancy at birth. For both sexes, mortality above age 60 years was higher than expected in 2020, with negative contributions of >1 year for men, but this unfavourable development was offset by a faster-than-expected mortality decline below age 60 years. In 2021, the same phenomenon is observed, but the increase in mortality between 60 and 80 years of age is greater. The risk of dying between the ages of 60 and 80 years is 16% higher than expected for women (+20% for men) and the mortality rate above 80 years is 14% higher (+44% for men).

**Table 2:** Life expectancy at birth observed in 2019–21, predicted for 2020–21 based on past trends, in the absence of COVID-19, and decomposition of the differences in life expectancy at birth between the observed and predicted estimates

	Observed			Predicted based on 1990–2019		Difference in e0 and contribution in difference by age*	
	2019	2020	2021	2020	2021	2020	2021
Life expectancy at birth (years)							
Male	63.3	63.3	62.8	63.4	63.6	−0.14	−0.80
Female	68.7	68.9	68.0	68.8	69.0	0.03	−1.02
Under-five mortality (5q0—‰)							
Male	40	34	32	45	43	0.76	0.77
Female	38	30	29	39	37	0.58	0.59
Mortality in older children and adolescents (10q5—‰)							
Male	8	7	5	8	8	0.04	0.12
Female	8	4	5	6	6	0.10	0.03
Premature adult mortality (_45_q_15_—‰)							
Male	314	314	306	316	316	0.15	0.42
Female	199	201	208	204	204	0.10	−0.04
Old-age mortality (_20_q_60_—‰)							
Male	637	683	754	629	627	−0.94	−1.83
Female	517	543	592	513	509	−0.67	−1.38
Mortality rate above age 80 (_∞_m_80_—‰)							
Male	187	200	251	174	174	−0.16	−0.29
Female	170	182	199	174	174	−0.08	−0.22

*The last two columns display the difference in life expectancy at birth between the observed and expected values (in years), and these differences are decomposed into the contributions of the different age-specific rates, using the method by Arriaga.^23^. Positive values in these last two columns refer to a decline in mortality, which was faster than expected based on trends in 1990–2019, while negative values refer to a slower progress than expected, or even an increase in mortality, possibly due to COVID-19 deaths.

As a result, favourable trends in mortality below the age of 60 years are no longer sufficient to compensate the excess mortality above the age of 60 years and life expectancy is reduced by 0.8 years for men and 1.02 years for women compared with expected levels. In other words, the loss of life expectancy in 2021 is explained exclusively by the sharp increase in a risk of dying above the age of 60 years. In children, youth or adults aged 15–59 years, the various direct or indirect effects of the pandemic (lockdowns, reductions in intervention coverage, etc) did not result in a loss in life expectancy.

We further decomposed the differences in life expectancy at birth between 2019 and 2020, and between 2020 and 2021, by the contribution of different age groups and two categories of causes of death: deaths identified as COVID-19 and all other deaths. From 2020 to 2021, the increase in mortality over age 60 years is attributable to deaths identified as COVID-19 and non-COVID-19 deaths (online supplemental figure S6). Between 2020 and 2021, more years are lost due to the increase in COVID-19-related mortality, for both sexes. The loss of years of life expectancy is concentrated in the 60–79 years age group, with little change in women in non-COVID-19 mortality. Among men, mortality in the age group 35–59 years from non-COVID-19 mortality seems to have improved when compared with 2020, but this could also reflect a more systematic coding of deaths as COVID-19 related (online supplemental table S4).

## Discussion

After over three and a half years since the beginning of the pandemic, the mortality burden due to COVID-19 across sub-Saharan Africa remains difficult to quantify. Taking advantage of a longstanding, rigorous death registration system from the city of Antananarivo, our study found several peaks of excess deaths in 2020–21, which were associated significantly with the waves of SARS-CoV-2 reported cases. By comparing trends during the prepandemic period (2016–19) and the pandemic one (2020–21), we estimated that all-cause excess mortality was 38.5 and 64.9 per 100 000 inhabitants in 2020 and 2021, respectively. Excess mortality reached over 50% during both the 2020 and 2021 waves. In sum, we estimated that there were 1179 excess deaths over the 2020–21 period, an estimate which is higher than the official 1069 COVID-19 deaths notified by the MoH for the entire country.^8^ The excess was even higher (1500) when excluding the excess deaths associated with the 2018–19 measles outbreaks, and twofold (2111) when no threshold was included in our calculation of relative change. This excess mortality resulted in a decline in life expectancy of 0.8 years in men and 1.0 years in women in 2021 but this decline is non-significant. The reduction in life expectancy was entirely attributable to increased risks of dying for both men and women above the age of 60 years and due to COVID-19 deaths rather than due to increases in other causes of death.

Interestingly, the death rate mainly preceded the SARS-CoV-2 positivity rate during the first wave of SARS-CoV-2, which may indicate an ‘unsuitable’ screening strategy and the use of a case definition^9 25^ for suspected cases where MoH and WHO guidelines differed.^26^ This may have led to a mismatch between waves because only few ‘true suspected cases’' were tested. In the second wave of COVID-19 (February–May 2021), the SARS-CoV-2 positivity rate preceded the waves of deaths. This may indicate an improvement in the screening strategy, enabling strong synchrony between laboratory data and virus circulation in Antananarivo, as previously found by Andriamandimby *et al*.^25^ Indeed, the National Influenza Centre at IPM continued to receive specimens for influenza in 2020 and 2021 and laboratory tests showed no circulation of influenza viruses from March 2020 to mid-July 2021.^27^ Our results also revealed that in the first half of 2020, the all-cause deaths in Antananarivo and national COVID-19 deaths were not synchronous. This may be explained by the fact that the first large SARS-CoV-2 outbreak occurred in the second highest populated city of Madagascar (Toamasina) in May 2020, followed a few weeks later by a major outbreak in Antananarivo (June 2020).^9 25^

In the capital city of Antananarivo, we estimated an excess mortality of 38.51 and 64.91 per 100 000 inhabitants in 2020 and 2021, respectively. These estimates are lower than estimates for Madagascar in a worldwide study using covariates pertaining both to the COVID-19 pandemic and to country-specific health-related metrics (125.4 per 100 000 inhabitants).^27^ At the level of sub-Saharan African countries, this excess death is comparable to the excess death estimated for Eritrea, Burkina Faso and Ghana.^27^ Compared with previous crises that have hit the capital city of Madagascar, the mortality shock observed in 2020–21 is less pronounced than other major crisis that have hit Antananarivo before, such as the urban famine and the resurgence of malaria in the mid-1980s. When comparing with the excess death in Antananarivo that occurred during the A(H1N1) pandemic in 2009, a similar impact on elderly people was observed.^14^

Our study is among the first to assess the impact of COVID-19 on the life expectancy of a sub-Saharan African population, based on local death registration systems. In contrast to many high-income countries, where reductions of more than an entire year of life expectancy at birth have been documented in 2020,^28^ we found almost no change for that year (−0.14 for men and +0.03 for women). A faster decline in mortality before age 60 years than expected based on past trends could have offset the unfavourable evolution of mortality beyond age 60 years. Countries where life expectancy at birth has decreased considerably include Russia, the USA, Italy and Spain^29^; where populations are older and where baseline mortality rates are much lower in comparison with African populations. Mortality in Madagascar is still highly concentrated in the youngest age groups, and life expectancy remains dominated by variations in the chances of survival among children. Yet, the year 2021 was characterised by a 16% increase in the probability of dying between the ages of 60 and 80 years for women and a 20% increase for men in Antananarivo. The mortality rate over the age of 80 years increased by 14% for women and 44% for men. As observed elsewhere,^30^ men appeared to have been more affected by the COVID-19 epidemic than women.

Compared with previous years, the excess mortality in Antananarivo was higher in 2021, reaching over 100% for 4 weeks. As a result, a substantial decrease in life expectancy at birth was observed, dropping by 0.8 and 1.0 years below the projected value for males and females, respectively. A similar situation was observed in Brazil where a decline in 2020 life expectancy at birth was estimated at 1.3 years in 2020 vs 1.8 years in the first 4 months of 2021.^31^ Several countries in Eastern Europe and the USA also witnessed sustained loss in life expectancy in 2021.^32^ Data on the impact of COVID-19 on life expectancy in other sub-Saharan African countries are needed to compare these estimates and shed light on the timing of the mortality shock in this region.

Our results highlight the granularity of Antananarivo’s BMH death records as a powerful source to assess the burden of infectious diseases and the strengths and weaknesses of the Malagasy health system to face major threats such as the COVID-19 pandemic. Indeed, the estimated excess mortality in 2020 and 2021 could be due to the difficulty of finding intensive care unit beds for severe infections,^33^ with mechanical ventilators and trained personnel, in addition to frequent disruptions in electricity supply observed regularly in LMICs.^34^ The SARS-CoV-2 Beta variant (B.1.351) circulated in Madagascar from February 2021, preceding the beginning of the peak of mortality in 2021. Results from blood donors confirmed the contribution of this variant in the second COVID-19 wave that started in early 2021.^35^ The Alpha, Beta and Delta variants of concern were known to be more transmissible and having caused more severe infections than the ancestral SARS-CoV-2.^36^ In Qatar, patients infected with the Beta variant had an increased risk of progressing to severe disease and death compared with patients infected with the Alpha variant (B.1.1.7).^37^ This could explain the higher excess of death observed in our study during the second wave when compared with the first one, combined with a vaccination strategy that was not effective at this time.^38^ Optimal monitoring of variants and more studies on their severity and virulence in the general population are needed to better understand their burden.

Our study has some limitations. First, the Ambohidratrimo district—where the referral centre for severe COVID-19 infections has been established at the Centre Hospitalier Universitaire Anosiala since 8 July 2020, totalling 442 244 inhabitants according to the 2018 census,^16^ does not fall in the six central districts of Antananarivo city nor the area covered by the BMH.^39^ This may have led to an underestimation of excess mortality using BMH data; although all deaths that occurred among residents of the five central districts should be reported to the BMH, irrespective of the place of the occurrence of the death. Moreover, during the first waves of the pandemic, the virology unit at IPM was mainly focused on the diagnosis of SARS-CoV-2 due to logistic constraints and a lack of human resources, and this may have resulted in an underdiagnosis of other respiratory viral infections responsible for other causes of death. Similarly, a decrease in consultations was observed at health centres during the first and second waves of the pandemic,^40^ which resulted in an under-reporting of other infectious diseases. Second, in our analysis, the estimation of excess mortality focused on the weeks when the excesses were most evident—using Farrington surveillance algorithm,^17^ which may induce an underestimation of mortality excess versus relative change method based on of P-scores.^18^ The comparison of our results with those of other countries may be biased because of this methodological choice. In other studies, negative binomial or Poisson regression modelling combined with Bayesian approaches or ensemble modelling techniques have been commonly used.^1 7 27^ In addition, between 2016 and 2020, Madagascar experienced other epidemics such as plague and measles that could have had an impact on the mortality of the population of Antananarivo.^5 6^ These epidemics raise our baseline mortality, although we also conducted a sensitivity analysis based on the average number of deaths per week during the pre-COVID-19 period without such peaks, revealing more excesses during the pandemic period.

## Conclusion

Our findings indicate that the mortality burden of the COVID-19 pandemic in Antananarivo has been much higher than previously estimated, with excess deaths for the capital city being higher than the official count of COVID-19-related deaths for the entire country. The excess mortality was concentrated in older adults, with little impact on mortality among children and young adults. Our study also highlights the importance of local sources of death notification and the need to expand this system to other major cities in the country and use it for real-time surveillance to inform health policy makers. Strengthening local death registration systems in LMICs would allow more precise monitoring of changes in cause-specific mortality associated with COVID-19 and other potential future pandemics.

## Data Availability

Data are available on reasonable request.
