# Supplementary material for: Excess mortality associated with the COVID-19 pandemic during the 2020 and 2021 waves in Antananarivo, Madagascar
Source: BMJ Glob Health. 2023 Jul 26;8(7):e011801. doi: 10.1136/bmjgh-2023-011801 (PMC10373673; doi:10.1136/bmjgh-2023-011801)
Supplement: Supplementary data [file bmjgh-2023-011801supp001.pdf]

Supplementary materials

Excess mortality associated with the COVID-19 pandemic during the 2020 and 2021 waves in Antananarivo, Madagascar

J.H. Rabarison MD[1*], J.M. Rakotondramanga MSc[2,3,4,5*], R. Ratovoson PhD[2#], B. Masquelier PhD[6], A.M. Rasoanomenjanahary MD[7], A. Dreyfus PhD[2], A. Garchitorena PhD[2,5], F. Rasambainarivo PhD[8,9], N.H. Razanajatovo PhD[1], S.F. Andriamandimby PhD[1], C.J.E. Metcalf PhD[8,10], V. Lacoste PhD[1], J-M. Heraud PhD[1q$#], P. Dussart PhD[1$]

Content

Supplementary Methods S1–S3

Supplementary Figures S1–S6

Supplementary Tables S1–S4

Supplementary Methods

Method S1. The Bureau Municipal d'Hygiène (BMH) of Antananarivo

This office was created in 1916 and covers five of the six central districts of Antananarivo-city, corresponding to the district of Antananarivo-Renivohitra. Its role has been to provide free consultations, conduct vaccination campaigns, manage malaria prophylaxis, and isolate patients with highly infectious diseases. Since 1921, the BMH has also been in charge of home verification of deaths. Historically, this system of death verification was motivated by recurrent plague epidemics but was maintained for the purpose of disease and outbreak surveillance. The 6th district (Ambohimanarina) does not fall in the area covered by the BMH for historical reasons; this locality was an autonomous urban commune under the 1st Republic, from 1958–72. Community and hospital deaths are recorded in a single register as they are reported to the BMH. These certificates are mandatory to obtain burial authorization. This system contributes to maintaining a high completeness of death registration, estimated at over 90% since the 1970s.[1]

Method S2: Relative change method based on P-scores

The expected deaths were derived from the period 2016 to 2019, and the corresponding relative change was computed as follows:[2]

$$Relative\ change\ (\%) = \frac{\{Reported\ deaths\} - \{Average\ expected\ deaths\}}{\{Average\ expected\ deaths\}} * 100.$$

A relative change of 100%, in a given week in 2020 (or 2021), would mean that the death count for that week was double from the expected death count.

Method S3: Mathematical background to cross-wavelet analyses

Cross-wavelet analyses were performed twice in order to detect and quantify the existing relationships between (1) the log-transformed of all-cause deaths in Antananarivo and national COVID-19 deaths (supplementary Figure S1A and S1C),[3] and (2) the transformed square root of SARS-CoV-2 test positivity rate by the IPM laboratory and all-cause death rate in Antananarivo, (Supplementary Figure S1B and S1D), as non-stationary time series. The concepts of cross-wavelet coherency and wavelet phase difference are natural generalizations of the basic wavelet analysis tools that enable us to appropriately deal and conclude with the time-frequency dependencies between two time series.[4]

Cross-Wavelet Transform (XWT) of two time series, $x(t)$ and $y(t)$ is defined as

$$W_{xy} = W_x W_y^*,$$

Where W_x and W_y are the wavelet transforms of x and y, respectively. The cross-wavelet power (XWP) is

$$(XWP)_{xy} = |W_{xy}|.$$

Here, we can interpret the wavelet power spectrum to depict the local variance of a time series; the cross-wavelet power of two time series depicts the local covariance between these time series at each time and frequency (the inverse form of scale/period). Furthermore, the cross-wavelet power informs us about a quantified indication of the similarity of power between two time series.

Then, given two time series $x(t)$ and $y(t)$, their complex wavelet coherency can defined as ρ_{xy} by:

$$\rho_{xy} = \frac{S(W_{xy})}{\left[S(|W_x|^2)S\left(|W_y|^2\right)\right]^{1/2}},$$

where S denotes a smoothing operator in both time and scale (period). Without the smoothing, coherency would be identically one at all scales and times. That needs an appropriate choice of parameters combination. So, Bartlett window was used both for smoothing in time and scale (period) direction, without a detrending technique but with a default method of generating surrogate time series, white-noise.[5]

Then, the complex wavelet coherency can be written in polar form, as $\rho_{xy} = |\rho_{xy}|e^{i\Phi_{xy}}$:[6]

- The absolute value of the complex wavelet coherency is called the wavelet coherency and is defined as,

$$Coherency = R_{xy} = \frac{|S(W_{xy})|}{\left[S(|W_x|^2)S\left(|W_y|^2\right)\right]^{1/2}},$$

with $0 \leq R_{xy} \leq 1$ across time and scale direction.

Thus, the cross-wavelet coherence of a complex wavelet is,

$$Coherence = Coherency^2$$

- The angle of the complex coherency is called phase-difference (phase lead of x over y), i.e.

$$\Phi_{xy} = \left(\frac{Im\left(S(W_{xy})\right)}{Re\left(S(W_{xy})\right)}\right).$$

Thus, a phase-difference of zero indicates that the time series move together at the specified time-frequency; if $\Phi_{xy} \in \left(0, \frac{\pi}{2}\right)$, then the series move in phase, but x leads y; if $\Phi_{xy} \in \left(-\frac{\pi}{2}, 0\right)$, then it is y that is leading x. A phase-difference of π (or –π) indicates an anti-phase relation; if $\Phi_{xy} \in \left(\frac{\pi}{2}, \pi\right)$, then y is leading; x is leading if $\Phi_{xy} \in \left(-\pi, -\frac{\pi}{2}\right)$. This is illustrated by arrows directions as in Figure 3.[6]

References

1 Masquelier B, Pison G, Rakotonirina J, *et al.* Estimating cause-specific mortality in Madagascar: an evaluation of death notification data from the capital city. *Popul Health Metr* 2019;**17**:8.
2 Serfling RE. Methods for Current Statistical Analysis of Excess Pneumonia-Influenza Deaths. *Public Heal Reports* 1963;**78**:494.
3 Mathieu E, Ritchie H, Rodés-Guirao L, *et al.* Coronavirus Pandemic (COVID-19). *Our World Data* 2020.
4 Aguiar-Conraria L, Soares MJ. The Continuous Wavelet Transform: A Primer. 2011. Available: https://econpapers.repec.org/RePEc:nip:nipewp:16/2011
5 Cazelles B, Chavez M, Berteaux D, *et al.* Wavelet analysis of ecological time series. *Oecologia* 2008;**156**:287–304.
6 Roesch A, Schmidbauer H. WaveletComp: Computational Wavelet Analysis. 2018. Available:

https://cran.r-project.org/package=WaveletComp

Supplementary Figures

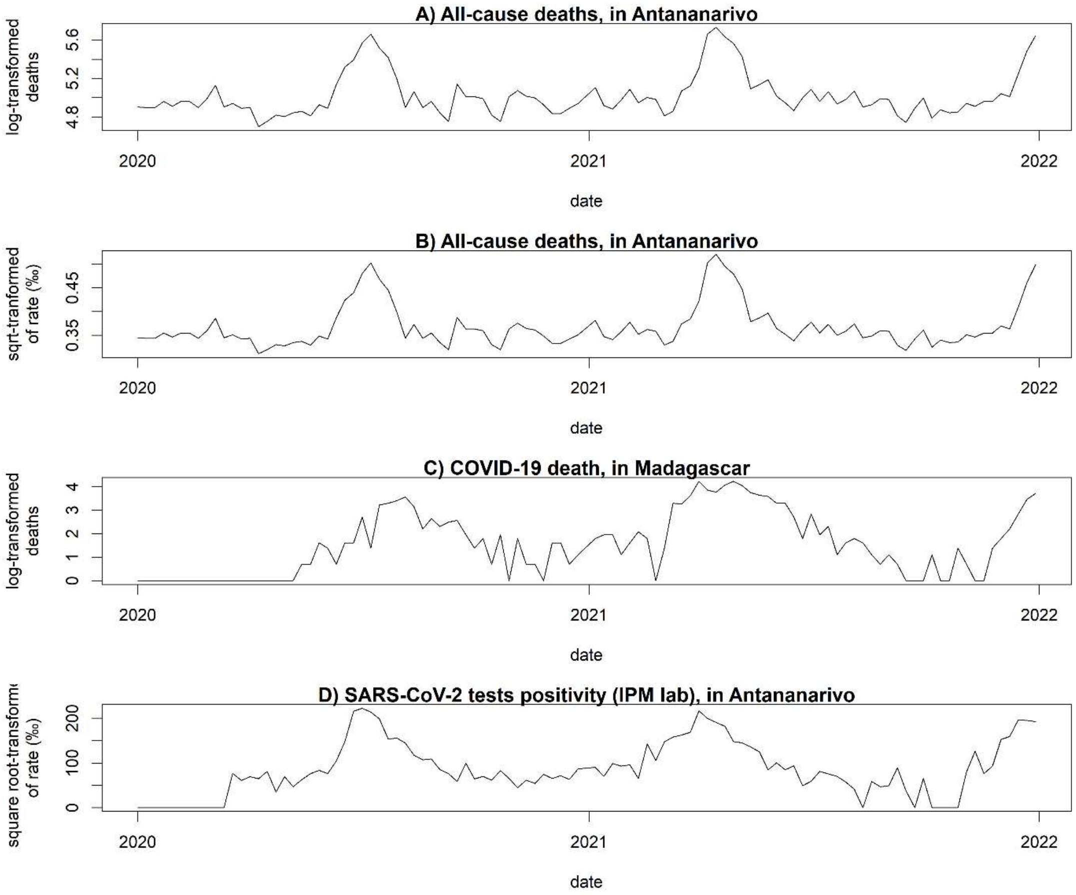

Figure S1. Temporal trends from 2020–21 of transformed data used for the wavelet analyses, cross-wavelet coherence and phase-difference: the log-transformed of all-cause deaths in Antananarivo and national COVID-19 deaths (panels A and C),[3] and the square root-transformed of SARS-CoV-2 test positivity rate by the IPM laboratory and all-cause death rate in Antananarivo (panels B and D).

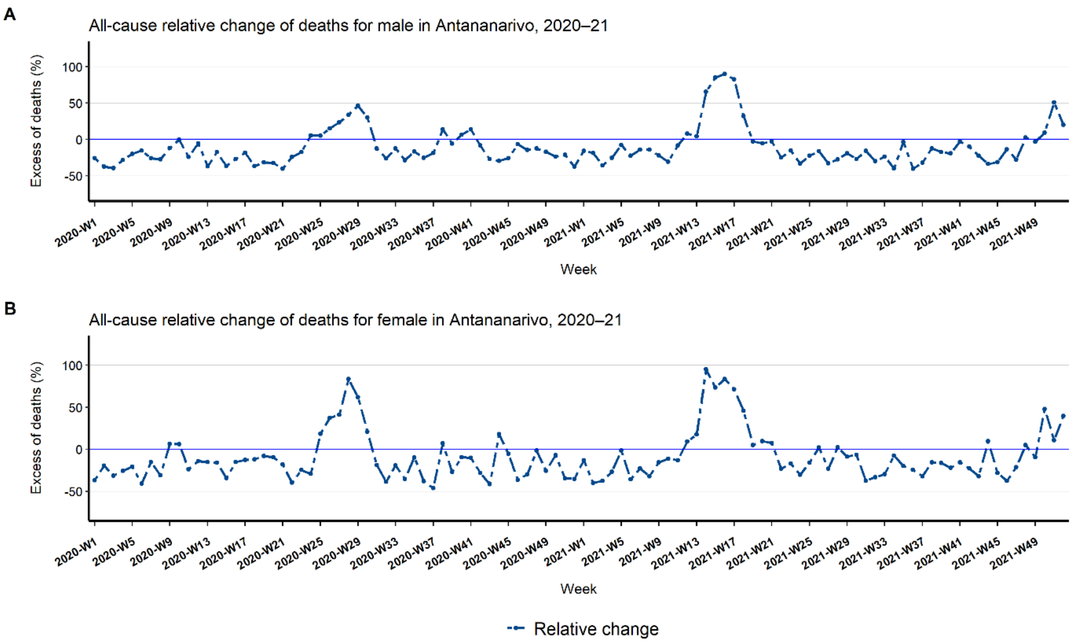

Figure S2: Weekly excess mortality by sex: for (A) Male and (B) Female deaths.

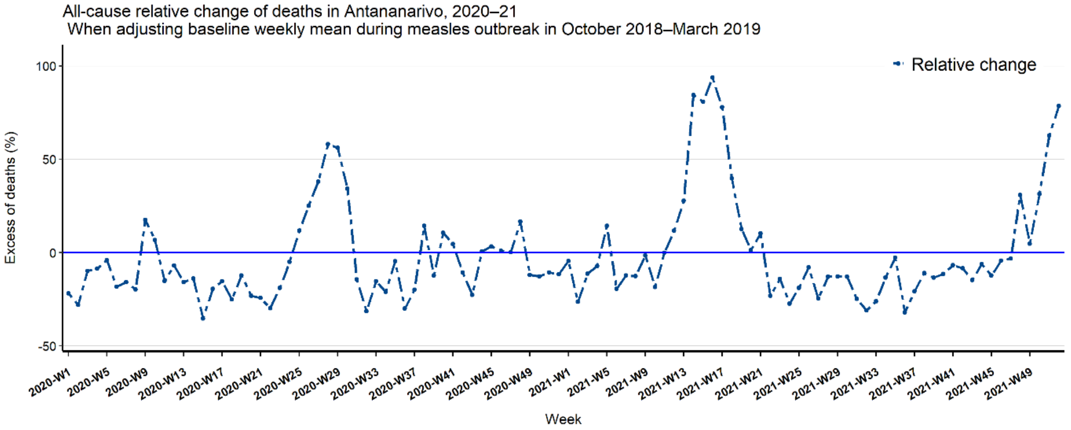

Figure S3. Weekly temporal trends, in five districts of Antananarivo from 2020–21. All-causes excess deaths were calculated using 2016–19 adjusted deaths as baseline (death from October 2018 to March 2019 was not considered due to measles outbreak affecting Antananarivo). Significant all-cause excess mortality was estimated during waves of COVID-19 in both 2020 and 2021 in Antananarivo: totalling 1,499 deaths for 32 weeks. (95 % CI is indicated in grey)

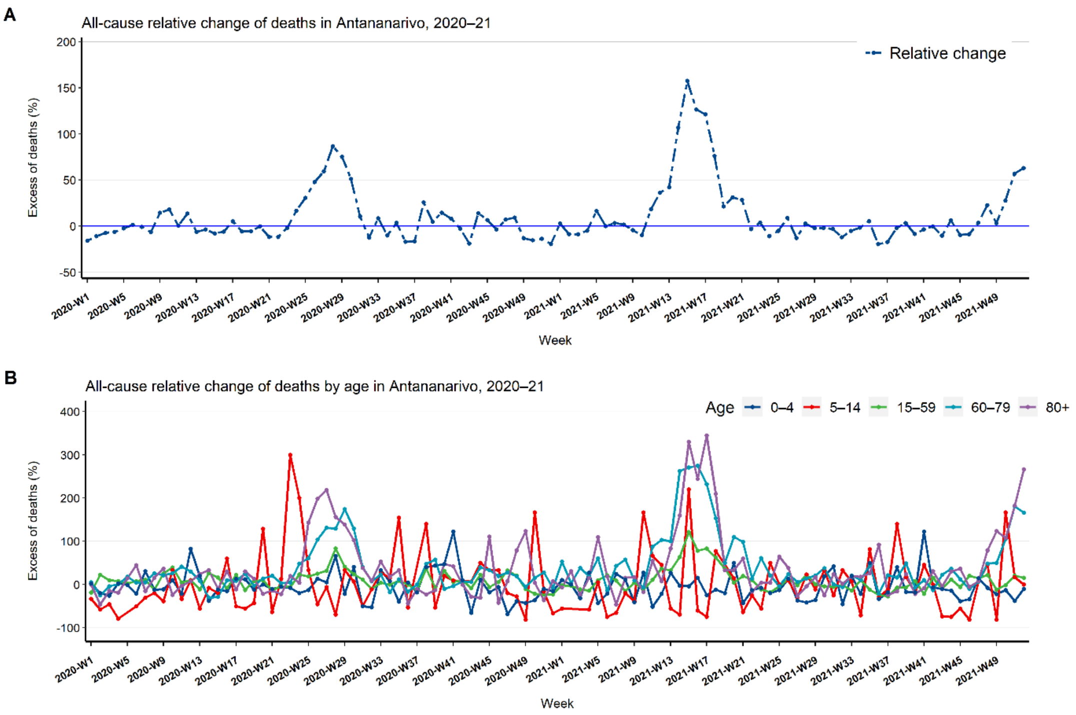

Figure S4. Weekly temporal trends using a relative change method based on P-scores, in five districts of Antananarivo from 2020–21. (**a**) All-cause excess mortality using 2016–19 crude deaths as baseline years, totalling 2,111 deaths for 51 weeks. (**b**) All-cause excess mortality by age group using 2016–19 crude deaths as baseline years; the excess mortality was estimated at over 200% for the elderly (80+) for each wave (no death record was observed for age group 5–14 age during one week in 2020 and two weeks in 2021). Supplementary Table S1 Supplementary materials representing weekly all-cause deaths by age group supports this figure.

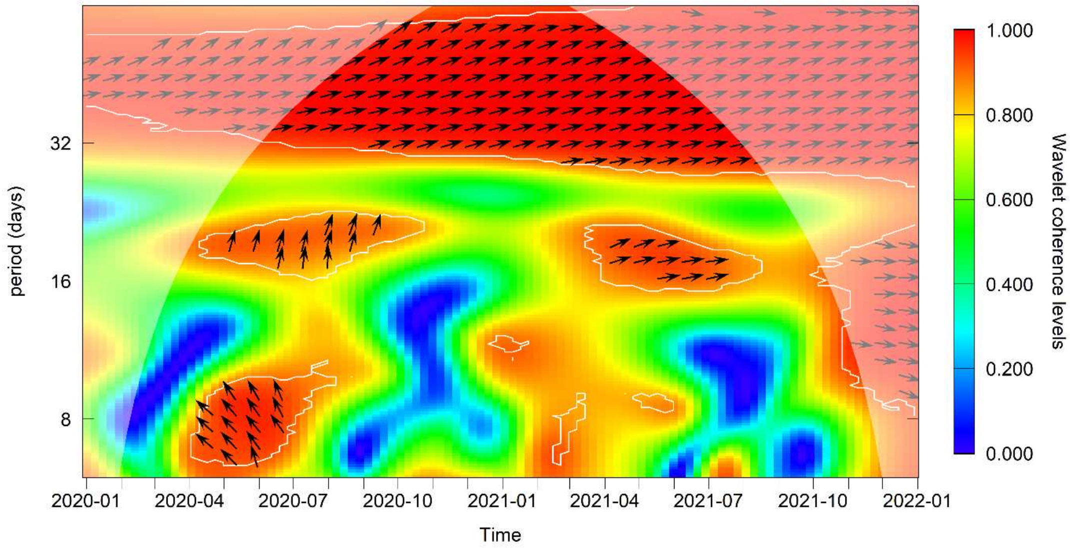

Figure S5: Cross-wavelet coherence and phase-difference of the all-cause deaths in Antananarivo and national COVID-19 deaths.[3] This figure shows that these two time series were out of phase, phase-differences $\in \left(\frac{\pi}{2}, \pi\right)$; and the weekly national COVID-19 deaths led the Antananarivo deaths. However, from the 2nd half of 2020 to September 2021, the all-cause deaths in Antananarivo were mainly in phase and led the national COVID-19 deaths, phase-differences $\in \left(0, \frac{\pi}{2}\right)$. The significant coherence level is ranked from blue–red colour scale, and statistically significant wavelet coherence is marked by the white contour plots when p-value < 0.05. This figure is supported by the Method S3, Figure S1A and S1C.

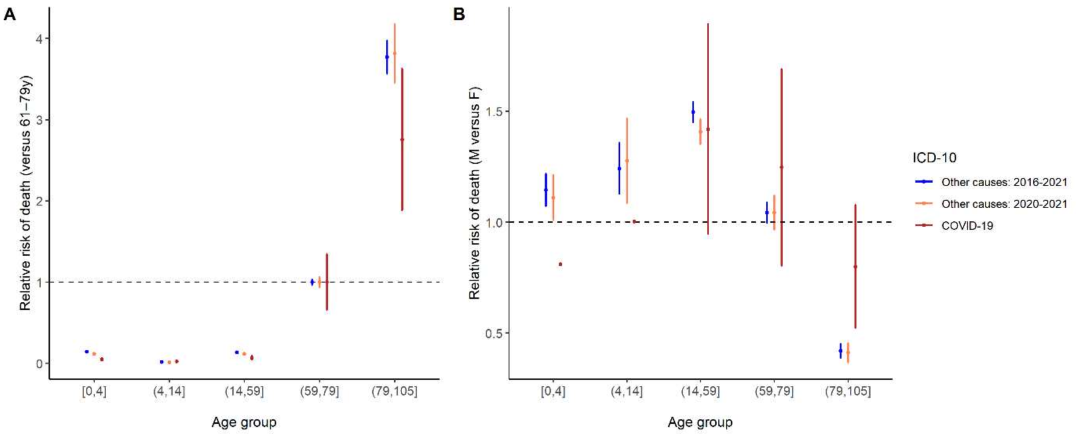

Figure S6. Relative risk ratios of COVID-19 and other causes of death, for each age group, (A) versus 60–79y, and (B) for Male (versus Female). The vertical bars represent confidence intervals. In 2020–2021, COVID-19 infection induced a significantly higher risk of death for the elderly aged 80+ compared to persons aged 60–79 years of age, with a risk ratio (RR) = 2.8 (IC95% 1.9–3.6) (panel A). A similar relative risk of dying from COVID-19 was observed for men and women in these two age groups, in comparison to other causes of deaths in the same period. That showed lesser risk for men 80+ years of age, RR = 0.4 (0.36–0.46) (panel B).

Supplementary Tables

Table S1: Weekly mortality data in Antananarivo, 2016–2021.

Year	Age	Mean	SD
**2016**	[0,4]	11.1	4.74
**2016**	(4,14]	3.02	1.63
**2016**	(14,59]	61.27	7.97
**2016**	(59,79]	33.17	5.87
**2016**	(79,105]	12.4	3.81
**2017**	[0,4]	11.73	3.75
**2017**	(4,14]	2.9	1.49
**2017**	(14,59]	62.31	12.15
**2017**	(59,79]	35.83	7.48
**2017**	(79,105]	12.25	4.41
**2018**	[0,4]	17	10.14
**2018**	(4,14]	3.92	2.51
**2018**	(14,59]	68.87	15.96
**2018**	(59,79]	37.69	8.43
**2018**	(79,105]	12.5	3.62
**2019**	[0,4]	16.12	5.9
**2019**	(4,14]	3.98	1.96
**2019**	(14,59]	69.23	11.44
**2019**	(59,79]	42.62	8.04
**2019**	(79,105]	15.19	4.29
**2020**	[0,4]	13.21	4.13
**2020**	(4,14]	3.18	1.82
**2020**	(14,59]	70.62	12.99
**2020**	(59,79]	46.96	19.25
**2020**	(79,105]	16.63	9.38
**2021**	[0,4]	12.75	3.92
**2021**	(4,14]	2.96	1.63
**2021**	(14,59]	71.21	15.09
**2021**	(59,79]	57.29	25.45
**2021**	(79,105]	19.1	9.49

Table S2: Weekly mortality data and excess deaths in Antananarivo, 2020-2021. Data shows estimated all-age excess mortality using positive lower bounds of relative change, the difference between weekly reported deaths in 2020–2021 and the upper bounds of 95%CI of the average expected deaths.

Week	Year	Reported deaths	Average expected deaths (2016–2019)			Death excesses by relative change
			Mean	CI Low	CI Up	
1	2020	135	160.25	134.22	186.28	-27.53
2	2020	134	150.25	110.81	189.69	-29.36
3	2020	134	144.50	82.02	206.98	-35.26
4	2020	143	152.50	121.41	183.59	-22.11
5	2020	136	139.25	111.80	166.70	-18.42
6	2020	143	141.00	83.80	198.20	-27.85
7	2020	143	144.25	111.40	177.10	-19.26
8	2020	134	143.50	101.07	185.93	-27.93
9	2020	147	128.50	115.22	141.78	3.68
10	2020	169	143.25	128.94	157.56	7.26
11	2020	135	134.25	91.96	176.54	-23.53
12	2020	140	123.25	97.29	149.21	-6.18
13	2020	133	142.00	104.82	179.18	-25.77
14	2020	134	138.75	121.79	155.71	-13.94
15	2020	110	119.50	68.64	170.36	-35.43
16	2020	116	123.25	102.57	143.93	-19.41
17	2020	124	118.00	89.36	146.64	-15.44
18	2020	122	129.50	95.91	163.09	-25.20
19	2020	127	134.25	123.59	144.91	-12.36
20	2020	129	129.50	91.10	167.90	-23.17
21	2020	123	139.25	116.03	162.47	-24.29
22	2020	138	156.25	115.99	196.51	-29.78
23	2020	133	135.75	107.33	164.17	-18.98
24	2020	170	145.75	112.54	178.96	-5.01
25	2020	204	156.00	129.31	182.69	11.66
26	2020	220	148.75	121.45	176.05	24.97
27	2020	262	164.25	138.58	189.92	37.95
28	2020	287	153.75	125.93	181.57	58.07
29	2020	249	142.00	124.52	159.48	56.13
30	2020	225	148.75	129.86	167.64	34.22
31	2020	181	164.00	116.39	211.61	-14.47
32	2020	134	153.50	111.23	195.77	-31.55
33	2020	158	145.50	104.14	186.86	-15.45
34	2020	134	149.25	128.65	169.85	-21.11
35	2020	143	138.25	126.46	150.04	-4.69
36	2020	127	152.75	124.10	181.40	-29.99
37	2020	116	138.75	132.47	145.03	-20.02
38	2020	171	136.00	122.44	149.56	14.33
39	2020	150	143.25	115.53	170.97	-12.27
40	2020	150	131.00	117.07	144.93	3.50
41	2020	147	135.75	131.00	140.50	4.63
42	2020	124	127.50	115.00	140.00	-11.43
43	2020	116	143.00	116.34	169.66	-31.63
44	2020	150	131.75	96.90	166.60	-9.96

45	2020	160	150.25	121.89	178.61	-10.42
46	2020	151	156.75	125.61	187.89	-19.63
47	2020	148	138.00	87.97	188.03	-21.29
48	2020	138	126.25	87.07	165.43	-16.58
49	2020	126	145.25	95.89	194.61	-35.26
50	2020	126	148.75	75.48	222.02	-43.25
51	2020	133	154.25	108.00	200.50	-33.67
52	2020	140	173.75	102.02	245.48	-42.97
1	2021	165	160.25	134.22	186.28	-11.42
2	2021	137	150.25	110.81	189.69	-27.78
3	2021	132	144.50	82.02	206.98	-36.23
4	2021	145	152.50	121.41	183.59	-21.02
5	2021	162	139.25	111.80	166.70	-2.82
6	2021	141	141.00	83.80	198.20	-28.86
7	2021	149	144.25	111.40	177.10	-15.87
8	2021	146	143.50	101.07	185.93	-21.48
9	2021	123	128.50	115.22	141.78	-13.25
10	2021	129	143.25	128.94	157.56	-18.13
11	2021	159	134.25	91.96	176.54	-9.93
12	2021	168	123.25	97.29	149.21	12.59
13	2021	202	142.00	104.82	179.18	12.73
14	2021	287	138.75	121.79	155.71	84.32
15	2021	308	119.50	68.64	170.36	80.79
16	2021	279	123.25	102.57	143.93	93.84
17	2021	261	118.00	89.36	146.64	77.98
18	2021	228	129.50	95.91	163.09	39.80
19	2021	163	134.25	123.59	144.91	12.48
20	2021	170	129.50	91.10	167.90	1.25
21	2021	179	139.25	116.03	162.47	10.18
22	2021	151	156.25	115.99	196.51	-23.16
23	2021	141	135.75	107.33	164.17	-14.11
24	2021	130	145.75	112.54	178.96	-27.36
25	2021	148	156.00	129.31	182.69	-18.99
26	2021	162	148.75	121.45	176.05	-7.98
27	2021	143	164.25	138.58	189.92	-24.71
28	2021	158	153.75	125.93	181.57	-12.98
29	2021	139	142.00	124.52	159.48	-12.84
30	2021	146	148.75	129.86	167.64	-12.91
31	2021	159	164.00	116.39	211.61	-24.86
32	2021	135	153.50	111.23	195.77	-31.04
33	2021	138	145.50	104.14	186.86	-26.15
34	2021	147	149.25	128.65	169.85	-13.45
35	2021	146	138.25	126.46	150.04	-2.69
36	2021	123	152.75	124.10	181.40	-32.20
37	2021	115	138.75	132.47	145.03	-20.71
38	2021	133	136.00	122.44	149.56	-11.08
39	2021	148	143.25	115.53	170.97	-13.44
40	2021	120	131.00	117.07	144.93	-17.20
41	2021	131	135.75	131.00	140.50	-6.76

42	2021	127	127.50	115.00	140.00	-9.28
43	2021	128	143.00	116.34	169.66	-24.55
44	2021	140	131.75	96.90	166.60	-15.96
45	2021	136	150.25	121.89	178.61	-23.86
46	2021	143	156.75	125.61	187.89	-23.89
47	2021	143	138.00	87.97	188.03	-23.95
48	2021	155	126.25	87.07	165.43	-6.30
49	2021	150	145.25	95.89	194.61	-22.92
50	2021	190	148.75	75.48	222.02	-14.42
51	2021	242	154.25	108.00	200.50	20.70
52	2021	283	173.75	102.02	245.48	15.28

Table S3. Estimated all-cause excess mortality in 2020–2021. Relative changes and expected deaths were calculated based on 2016–2019 baseline mortality adjusted by deaths during measles outbreaks (October 2018 to March 2019).

Year	Relative change level (%)	Nb. of weeks of excess	Adjusted expected deaths (2016–2019)[1]		Reported deaths (2020–2021)		Sum death excesses
			Mean	Range (min-max)	Mean	Sum	
2020	< 50	14	139	114-164	174	2,442	297
2020	≥ 100	2	148	142-154	268	536	195
2021	< 50	10	129	114-139	177	1,767	272
2021	≥ 100	6	132	118-150	277	1,66	736
Total		32	137	114-164	200	6,405	1,500

[1] Expected deaths were adjusted with deaths occurring during measles outbreak (October 2018-March 2019).
Nb=number

Table S4: Contribution of deaths identified as COVID-19 and those associated with other causes of deaths to changes in life expectancy at birth between 2019 and 2020, and between 2020 and 2021.

Age group	Males: e0(2020) – e0(2019)		Females: e0(2020) – e0(2019)		Males: e0(2021) – e0(2020)		Females: e0(2021) – e0(2020)	
	**Covid-19 deaths**	**All other causes**	**Covid-19 deaths**	**All other causes**	**Covid-19 deaths**	**All other causes**	**Covid-19 deaths**	**All other causes**
0–1	0.00	0.22	-0.01	0.07	0.00	0.01	0.00	0.06
1–4	0.00	0.19	0.00	0.44	0.00	0.12	0.00	0.03
5–14	0.00	0.08	0.00	0.21	0.00	0.05	0.00	-0.06
15–34	-0.02	0.04	-0.02	0.04	-0.04	0.08	-0.03	0.09
35–59	-0.13	0.27	-0.11	0.09	-0.31	0.62	-0.27	0.13
60–79	-0.17	-0.30	-0.19	-0.10	-0.48	-0.24	-0.55	-0.11
80+	-0.02	-0.04	-0.03	-0.09	-0.06	-0.09	-0.14	0.00
**Total**	**-0.35**	**0.45**	**-0.36**	**0.66**	**-0.90**	**0.55**	**-0.99**	**0.13**

The e_{xt} is the residual at age x and time t.
